# Supplementary material for: Targeted DNA methylation in vivo using an engineered dCas9-MQ1 fusion protein
Source: Nat Commun. 2017 Jul 11;8:16026. doi: 10.1038/ncomms16026 (PMC5508226; doi:10.1038/ncomms16026)
Supplement: Supplementary Information [file ncomms16026-s1.pdf]

File name: Supplementary Information
Description: Supplementary figures and supplementary tables.

File name: Peer review file
Description:

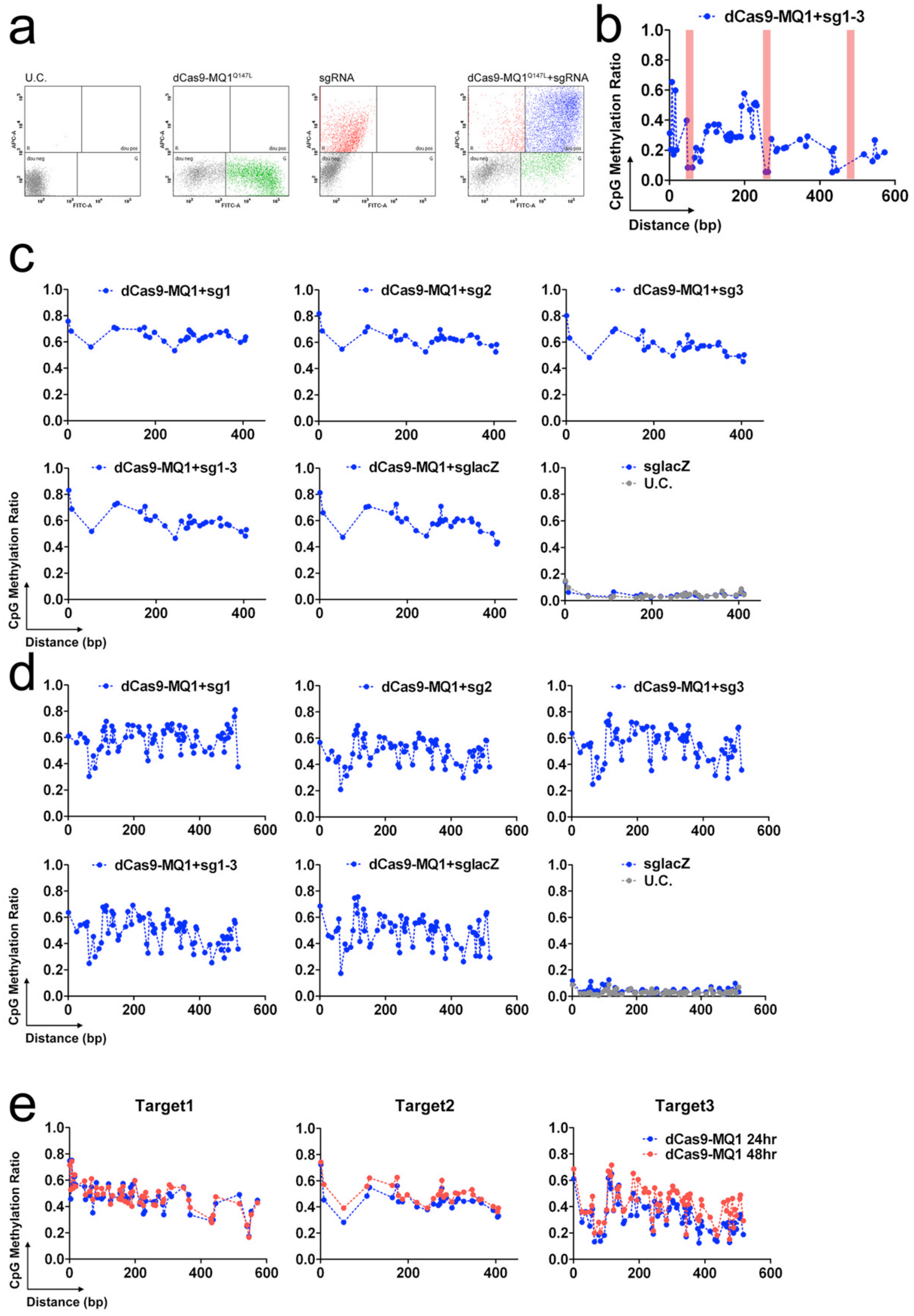


**Supplementary Figure 1. dCas9-MQ1 fusion protein induces *de novo* methylation.**

(**a**) Gating strategy for flow cytometry. The dCas9-MQ1 and its mutant variants plasmids express GFP,

and sgRNA plasmids express RFP621. FITC and RFP double positive cells were collected. (**b**) CpG methylation activity of dCas9-MQ1 at *HOXA5* CpG island. Graphs show the fraction of methylated CpGs (y axis) at different CpG sites along the length of the CpG island (x axis) between *HOXA5* and *HOXA6* loci. Red bars indicate the locations of *HOXA5* sgRNA binding sites. (**c** and **d**) CpG methylation activities of dCas9-MQ1 at *HOXA5* CpG island Target 2 (off-target) and Target 3 (off-target). Target 1 and 2 are located at the same CpG island (see Figure 1). Target 2 is 783 bp downstream of Target 1. Target 3 is located in the *HOXA4* CpG island. In the graphs above, sg1, *HOXA5* sgRNA1; sg2, *HOXA5* sgRNA2; sg3, *HOXA5* sgRNA3; sg1-3, the combination of *HOXA5* sgRNA1, sgRNA2, and sgRNA3. **(e**) Methylation pattern induced by dCas9-MQ1 fusion protein over time. Graphs show the fraction of methylated CpGs (y axis) at different CpG sites along the length of CpG island (x axis) at the indicated Target sites and indicated times after transfection.

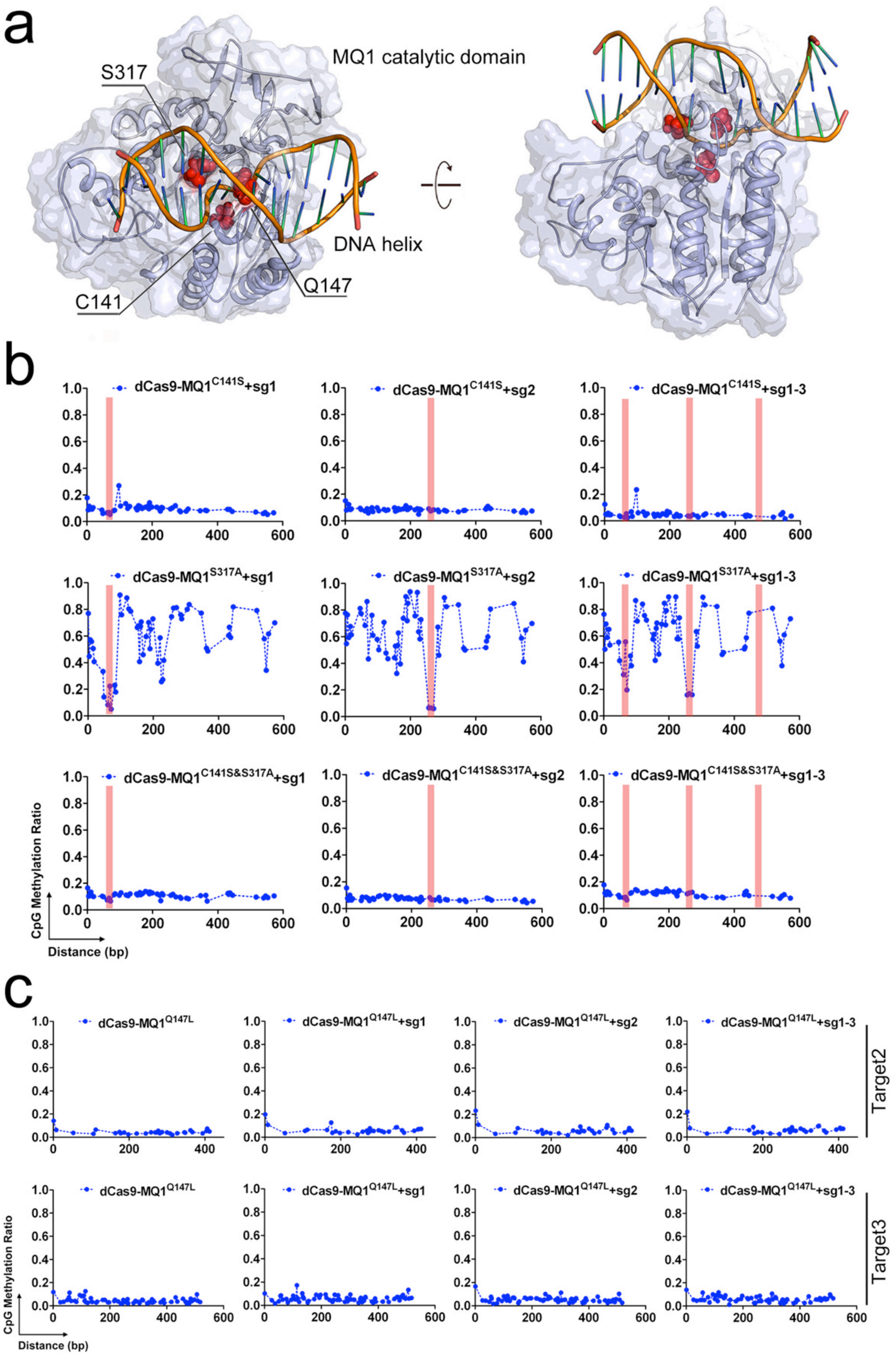


**Supplementary Figure 2. CpG methylation patterns induced by dCas9-MQ1 mutant fusion proteins.**

(**a**) Structural model of the MQ1 catalytic domain complexed with DNA. The MQ1 catalytic domain is

shown as a gray cartoon with surface rendering with a bound DNA double helix (gold ribbon). Mutations made in the current study (C141, Q147, and S317) are shown as spheres in red. (**b**) CpG methylation patterns induced by dCas9-MQ1$^{C141S}$, dCas9-MQ1$^{S317A}$, and dCas9-MQ1$^{C141S\&S317A}$. Graphs show the fraction of methylated CpGs (y axis) at different CpG sites along the length of the CpG island (x axis) at Target 1 (on-target) between *HOXA5* and *HOXA6* loci. Red bars indicate the locations of three sgRNAs binding sites. PAM is located at right side of red bar. (**c**) CpG methylation patterns at Target 2 and Target 3 in the presence of dCas9-MQ1$^{Q147L}$. Graphs show the fraction of methylated CpGs (y axis) at different CpG sites along the length of the CpG island (x axis) at Target 2 (off-target) and Target 3 (off-target).

a

| | dCas9-MQ1$^{Q147L}$ plasmid (ug) | sgRNA plasmid (ug) | Cell viability | Peak CpG methylation ratio |
|---|---|---|---|---|
| #1 | 0.5 | 1.0 | 77.2% | 42.7% |
| #2 | 1.0 | 1.0 | 81.2% | 33.5% |
| #3 | 1.0 | 2.0 | 73.4% | 60.4% |
| #4 | 2.0 | 2.0 | 68.0% | 59.1% |
| #5 | 4.0 | 4.0 | 64.0% | 61.5% |
| #6 | 0.0 | 0.0 | 85.4% | 3.0% |

b

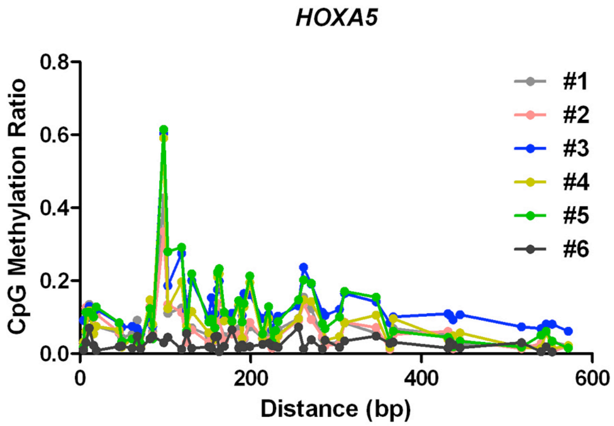


**Supplementary Figure 3. Dose-response experiment of dCas9-MQ1$^{Q147L}$.**

(**a**) Experimental design and plasmid concentrations transfected into HEK293T cells. The DNA amount was designed for each well of a 6-well plate. Cell viability was assessed by fluorescent activated cell sorting (FACS) during cell harvest at 24 hr. post transfection. Peak CpG means CpG site is located at 24 bp downstream from the *HOXA5* sgRNA1 binding site. Its methylation ratio was obtained via Nextseq. (**b**) CpG methylation patterns at the *HOXA5* locus in the presence of various amounts of dCas9-MQ1$^{Q147L}$ and sgRNA plasmids. Graph shows the fraction of methylated CpGs (y axis) at different CpG sites along the length of the CpG island (x axis).

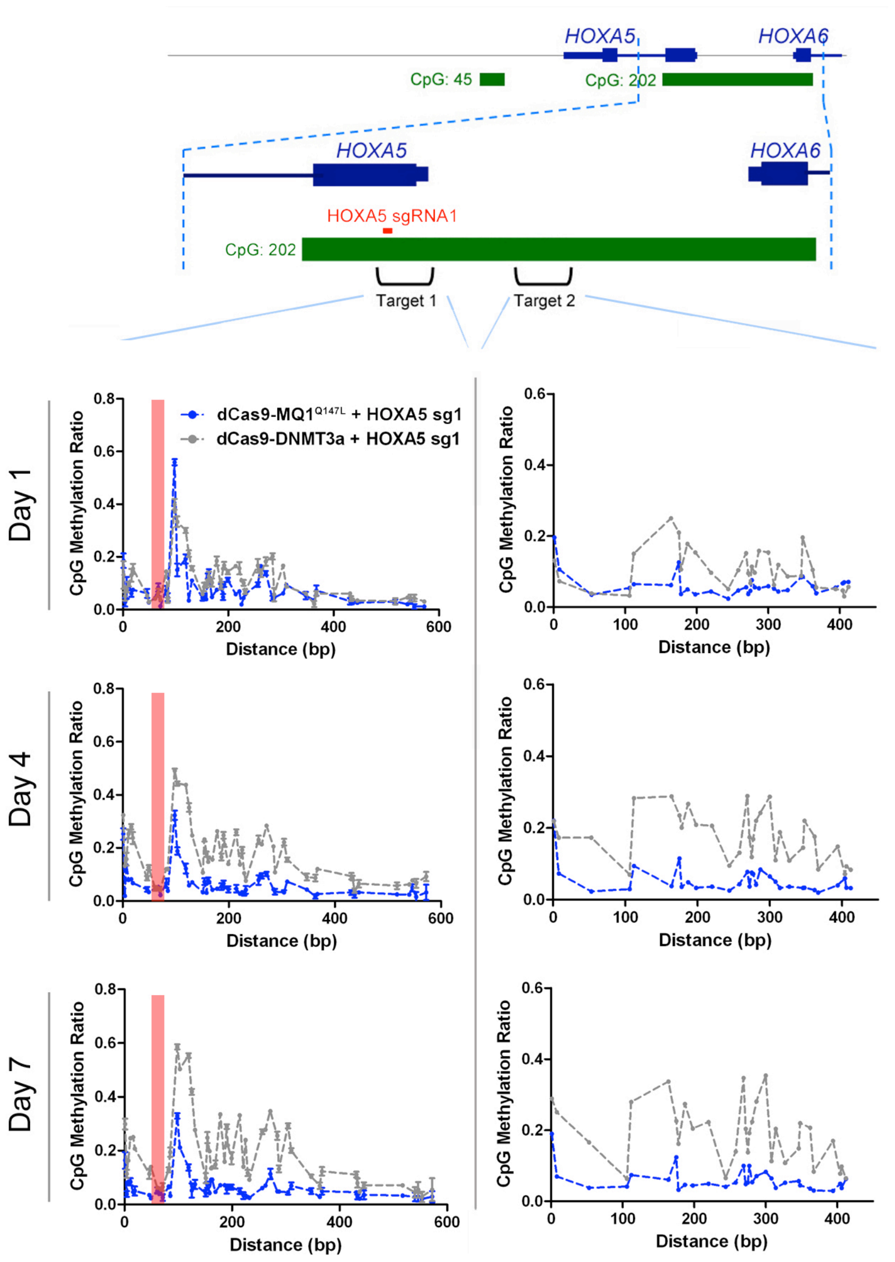


**Supplementary Figure 4. Time-course experiments of dCas9-MQ1$^{Q147L}$ and dCas9-DNMT3a at the *HOXA5* locus.**

Schematic illustration of the position of *HOXA5* targeted CpG island and *HOXA5* sgRNA1 binding sites in red. Target 1 amplicon covered the sgRNA1 binding site, while target 2 amplicon is located about 1.5 kb away from sgRNA binding site. Graphs show the fraction of methylated CpGs (y axis) at day 1, day 4, and day 7 post transfection at different CpG sites along the length of the CpG island (x axis). Red bars indicate the locations of sgRNAs binding sites. PAM is located at right side of red bar. CpG methylation ratio at target 1 was calculated based on biological triplicates. Error bars represent mean ± s.d.. CpG methylation ratio at target 2 was showed based on single experiment.

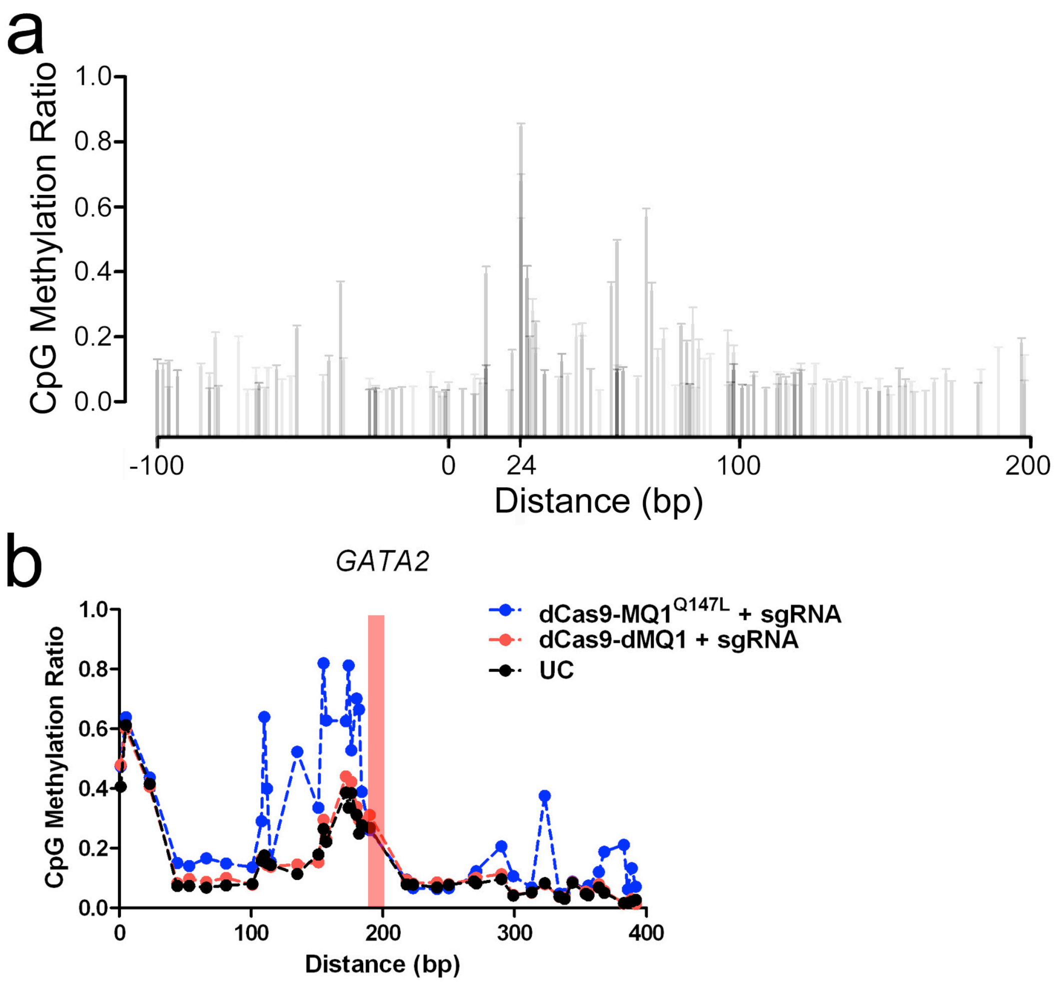


**Supplementary Figure 5. Summary profile of CpG methylation ratio.**
(**a**) Summary profile of dCas9-MQ1$^{Q147L}$ activity in ~300 bp region (from -100 to +200) is based on CpG methylation activities of *HOXA5* sg1, *HOXA4* sg1, *HOXA4* sg2, and *EYA4* sgRNA. The sgRNA binding sites are from -19 to 0. PAM is from +1 to +3. CpG methylation ratio was calculated based on Nextseq from biological triplicates. Error bars represent mean ± s.e.m. of biological triplicates. (**b**) CpG methylation patterns induced by dCas9-MQ1$^{Q147L}$ at *GATA2* locus. Graphs show the fraction of methylated CpGs (y axis) at different CpG sites along the length of the CpG island (x axis). Red bars indicate the locations of three sgRNAs binding sites. PAM is located at right side of red bar. Error bars represent mean ± s.e.m. of biological duplicates.

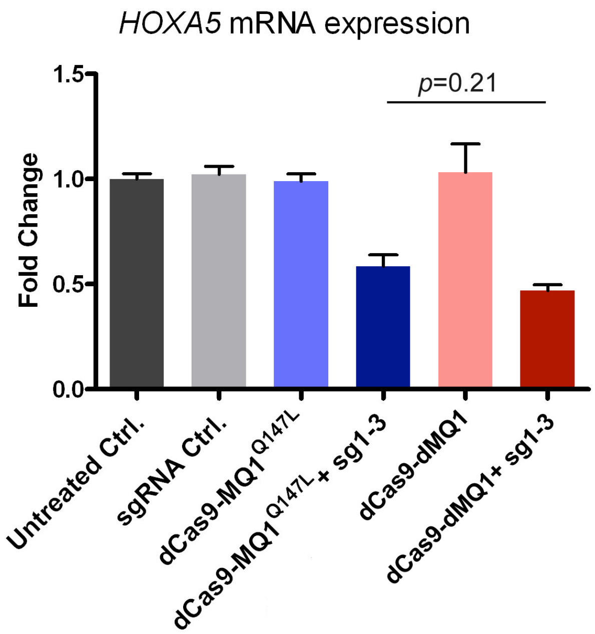


**Supplementary Figure 6. RT-qPCR of *HOXA5* gene expression.**

Error bars represent mean ± s.e.m. of biological triplicates. $p$ value from two-tailed $t$-test. 18s RNA was selected as internal control. sgRNA sequences are provided in **Supplementary Table 1**. Primers are provided in **Supplementary Table 2.**

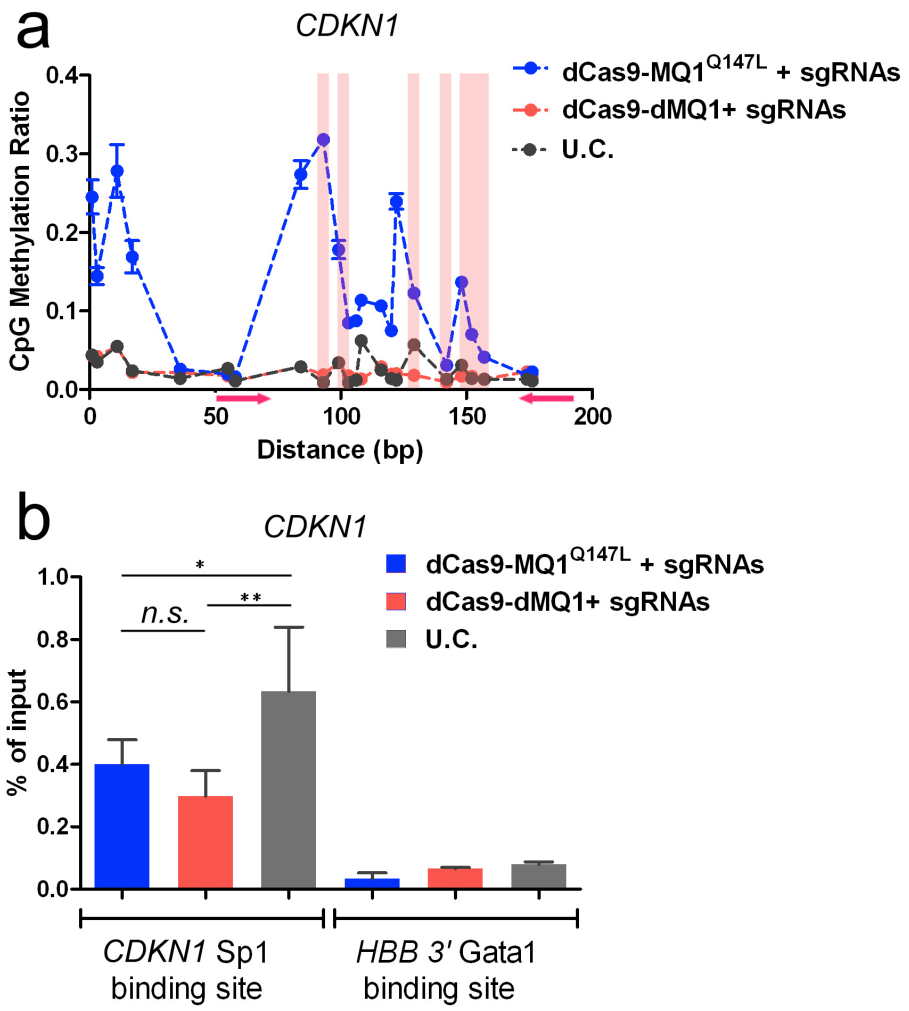


**Supplementary Figure 7. Targeted methylation of SP1 binding sites at CDKN1 promoter.**
(**a**) Methylation pattern (y axis) of different CpG along the length of target site (x axis) at CDKN1 promoter. Red arrows indicate the location of two sgRNA binding sites and toward to PAM. Red shadows indicate transcription factor SP1 binding sites. Error bars represent mean ± s.e.m. of biological triplicates**.**
(**b**) Anti-SP1 ChIP-qPCR was performed using cells in (**a**). Transcription factor GATA1 binding site at the 3' UTR of hemoglobin subunit beta (HBB) was selected as negative control of ChIP-qPCR. Error bars represent mean ± s.e.m. of experimental triplicates. * $p < 0.05$, ** $p < 0.01$, n.s. Not significant (two-tailed *t*-test).

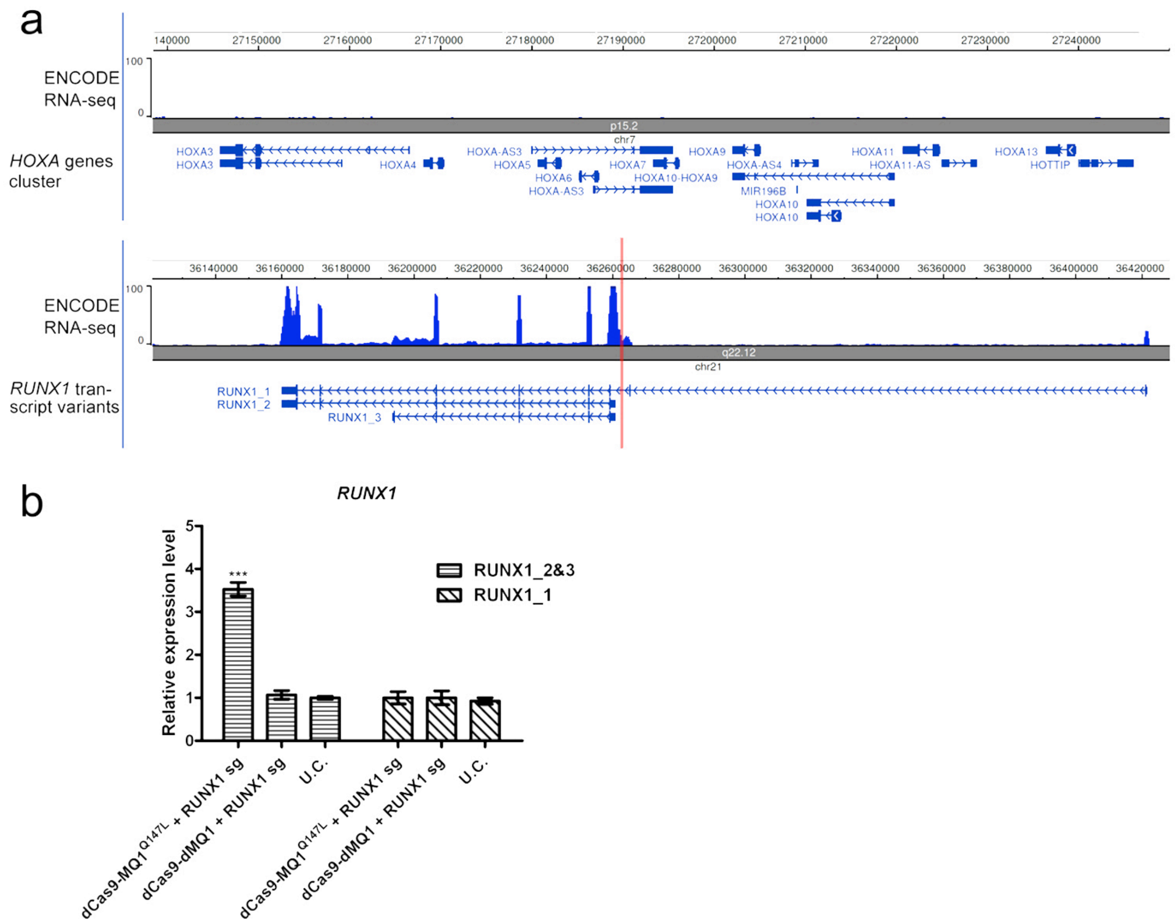


**Supplementary Figure 8. RT-qPCR of *RUNX1* gene expression from transcript variants**

(**a**) Schematic illustration of *HOXA* gene cluster and three transcript variants of *RUNX1* gene. Transcript details from RUNX1_1 (NM_001754.4), RUNX1_2 (NM_001001890.2), and RUNX1_3 (NM_001122607.1) showed. ENCODE RNA-seq of K562 cell was provided and *RUNX1* sgRNA binding sites was highlighted in red. (**b**) Error bars represent mean ± s.d. of experimental tetraplicates. *** $p < 0.001$, $p$ value from two-tailed $t$-test. β-actin was selected as internal control. U.C. indicates untransfected control. sgRNA sequences were provided in **Supplementary Table 1**. RT-qPCR primers were provided in **Supplementary Table 2.**

**a**

| | dCas9-MQ1$^{C141S}$ D1 | dCas9-MQ1$^{C141S}$ D2 | dCas9-MQ1$^{C141S}$ D4 | dCas9-MQ1$^{Q147L}$ D1 | dCas9-MQ1$^{Q147L}$ D2 | dCas9-MQ1$^{Q147L}$ D4 | dCas9-MQ1 D1 | U.C. D1 | U.C. D4 |
|---|---|---|---|---|---|---|---|---|---|
| dCas9-MQ1$^{C141S}$ D1 | 1.00 | 0.86 | 0.72 | 0.73 | 0.49 | 0.68 | 1.0E-75 | 0.90 | 0.96 |
| dCas9-MQ1$^{C141S}$ D2 | | 1.00 | 0.85 | 0.87 | 0.60 | 0.81 | 1.9E-74 | 0.95 | 0.90 |
| dCas9-MQ1$^{C141S}$ D4 | | | 1.00 | 0.99 | 0.74 | 0.96 | 1.4E-72 | 0.81 | 0.75 |
| dCas9-MQ1$^{Q147L}$ D1 | | | | 1.00 | 0.73 | 0.95 | 7.8E-73 | 0.82 | 0.76 |
| dCas9-MQ1$^{Q147L}$ D2 | | | | | 1.00 | 0.78 | 1.9E-70 | 0.57 | 0.52 |
| dCas9-MQ1$^{Q147L}$ D4 | | | | | | 1.00 | 4.8E-73 | 0.77 | 0.71 |
| dCas9-MQ1 D1 | | | | | | | 1.00 | 1.3E-74 | 3.2E-75 |
| U.C. D1 | | | | | | | | 1.00 | 0.94 |
| U.C. D4 | | | | | | | | | 1.00 |

*p* value of pairwise comparison by two-tail t-test

**b**

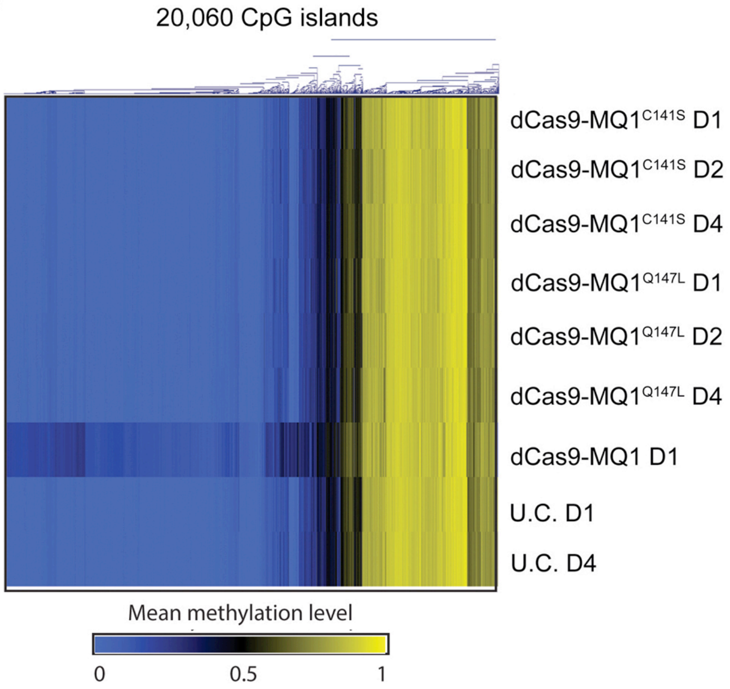


**Supplementary Figure 9. dCas9-MQ1$^{Q147L}$ induced specific CpG methylation without altering global methylation.**

(**a**) *p* value of pairwise comparison of two-tailed *t*-test among groups. (**b**) Genome-wide CpG methylation heat map using RRBS. About 72% of genome-wide CpG islands (20,060 out of 27,719) covered by all groups were analyzed.

a

| | Sequence | Score | Mismatch | Genes | Locus location |
|---|---|---|---|---|---|
| #1 | GGCGGTCGCAGCCGGGCGAGGGG | 6.2 | 2MMs [4:10] | NM_173814 | chr15:+56035087 |
| #2 | GCCTGTCTCTGCAGGGCGAGCGG | 1.1 | 3MMs [2:8:13] | | chr14:+35761950 |
| #3 | GGCTTTCGCGGCCGGGCGGGCAG | 0.9 | 3MMs [5:10:19] | | chr7:+45040123 |
| #4 | GTCTCTCGGCGCCGGGCGAGTGG | 0.8 | 4MMs [2:5:9:10] | | chr1:+226496433 |
| #5 | GGCTGTGGCAGGCGGGCGAGCGG | 0.8 | 3MMs [7:10:12] | | chr16:-67048011 |
| #6 | GGAAGGCGCCGCCGGGCGAGGAG | 0.8 | 4MMs [3:4:6:10] | NM_001278204 | chr5:-113698107 |
| #7 | GGCTGTGACTGCCGGGGGAGAAG | 0.7 | 3MMs [7:8:17] | | chr22:-30826124 |
| #8 | GACTGACGCTGCTGGGCGAGGGG | 0.7 | 3MMs [2:6:13] | | chr10:-83633874 |

b

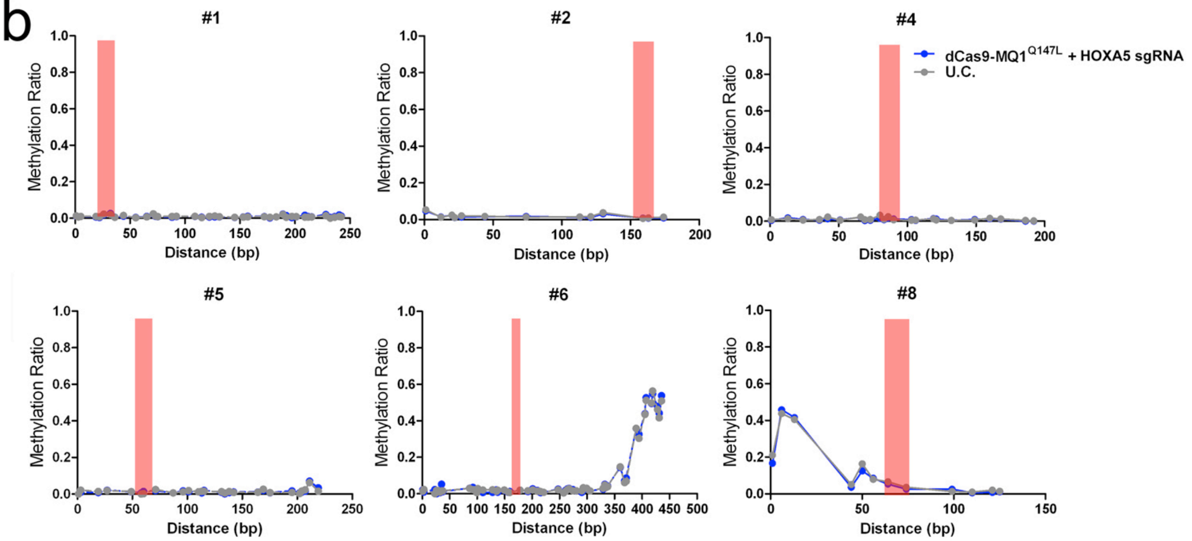


**Supplementary Figure 10. dCas9-MQ1$^{Q147L}$ showed no methylation activities at sgRNA similar sites.**

(**a**) The summary of the top eight off-target sites of *HOXA5* sg1 predicted using MIT CRISPR Design website (http://crispr.mit.edu/). (**b**) Methylation pattern (y axis) of different CpG along the length of target site (x axis) at different potential off-target loci. Red bars indicate the locations of similar sgRNAs binding sites. Off-target #3 and #7 loci are failed in bisulfited PCR amplification.

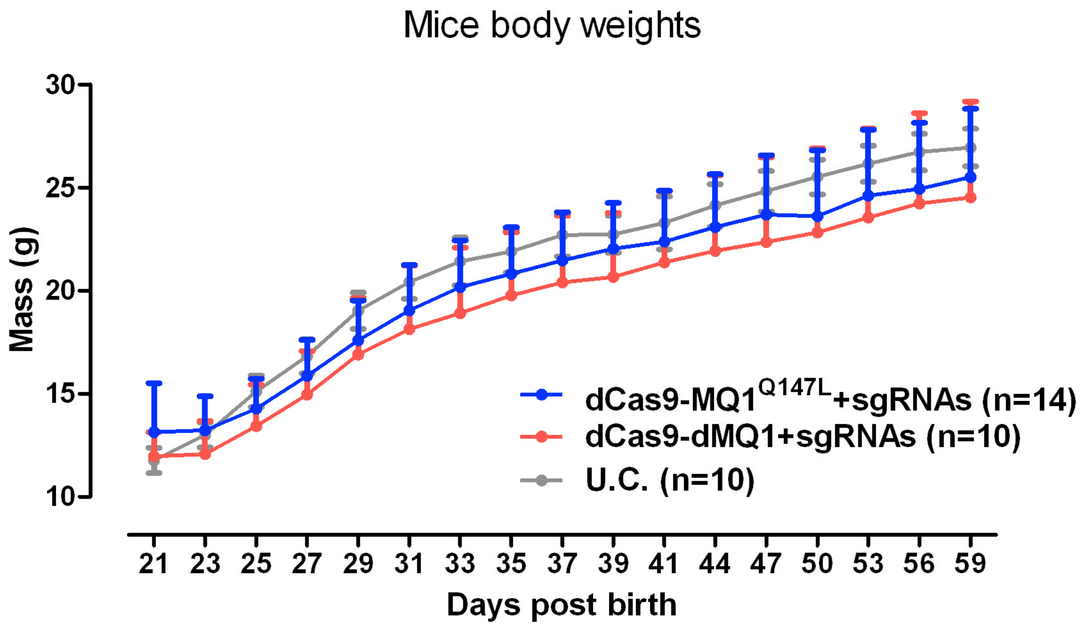


**Supplementary Figure11. Mice body weights post birth.**

Error bars represent mean ± s.e.m. of mice body weights. n indicates the mice number used for statistic.

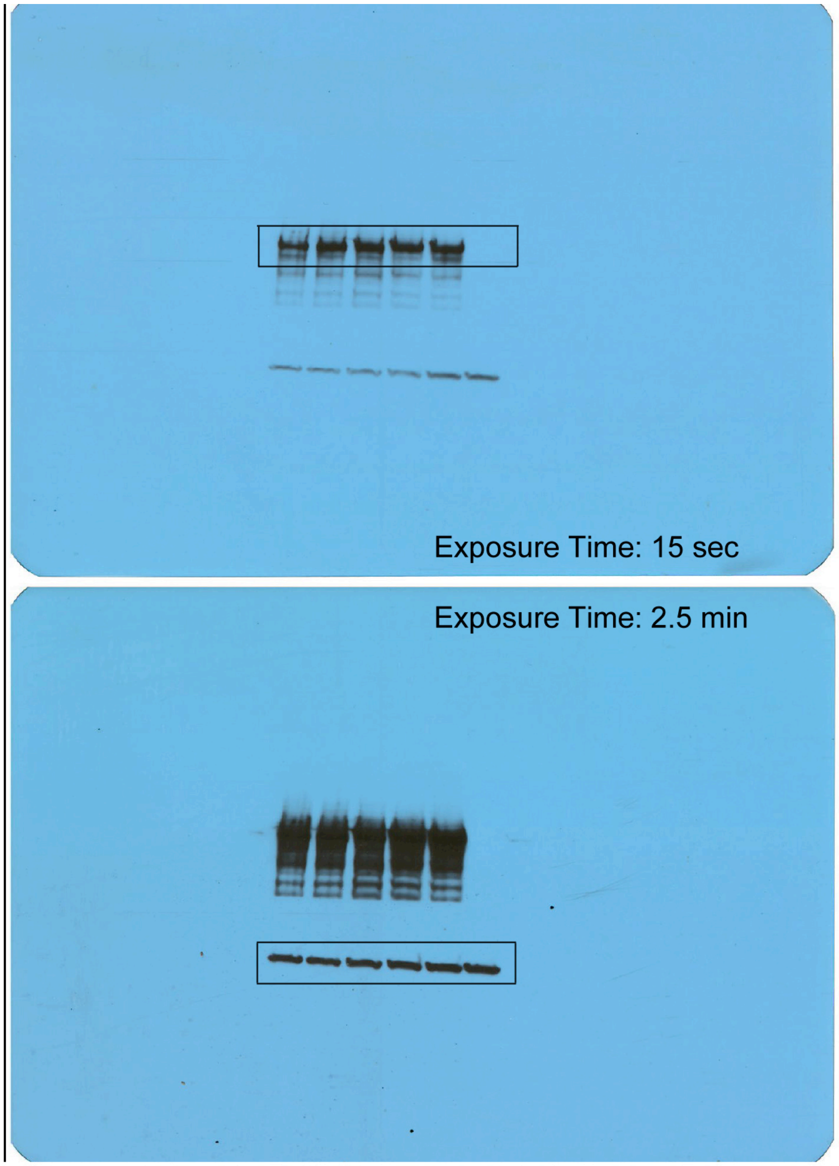


**Supplementary Figure12. Full blot images for protein expression of various dCas9-MQ1 mutant fusions.**

Black block indicates cropped regions in Figure 2b.

**Supplementary Table 1. sgRNAs target sequences.**
The tri-nucleotides highlighted in red are PAM sequences.

| Targets | Sequences |
|---|---|
| ***HOXA5 sg1*** | GGCTGTCGCTGCCGGGCGAGGGG |
| ***HOXA5 sg2*** | GTAGCCGTAGCCGTACCTGCCGG |
| ***HOXA5 sg3*** | GCACAATTTATGATGAATTATGG |
| ***LacZ*** | TCGTTTTACAACGTCGTGACTGG |
| ***HOXA4 sg1*** | TGGGCCCTTGGCTTGCGCCGGGG |
| ***HOXA4 sg2*** | GCCGGGGGCTGCTCGGGCTGGGG |
| ***EYA4*** | GCTGACCCAGAGTCCTTCGGAGG |
| ***GATA2*** | GAGCGGGGTTGGCATAGTAGGGG |
| ***HOXA11*** | TGGGGTTCCCGGCTCCCCGGCGG |
| ***HOXA13*** | GCTCATGAATTGGCCTTAGCTGG |
| ***RUNX1*** | GGGACTGCGCGCGGCGTCCAGGG |
| ***CDKN1 sg1*** | TGGCTCGGCGCTGGGCAGCCAGG |
| ***CDKN1 sg2*** | TCAGCTGGCGCAGCTCAGCGCGG |
| ***mH19_5.1a*** | GACGTCTGCTGAATCAGTTGTGG |
| ***mH19_5.1b*** | GTGGGGGGGCTCTTTAGGTTTGG |
| ***mH19_5.2*** | AATCAGTTGCAATCCGTTTTAGG |
| ***mH19_5.3*** | CCCCCAAGTTGGCAGCATTTGGG |
| ***mH19_5.4*** | CTCTCCACGCTGTGCAGATTTGG |

**Supplementary Table 2. Primers used in molecular cloning, bisulfite PCR, RT-qPCR experiments, and for site-directed mutagenesis.**

| Names | Sequences |
|---|---|
| pLV-MQ1 F | GCGCCCTAGGGACAGCAAAGTGGAGAACAAAACA |
| pLV-MQ1 R | GCGCCCTAGGGCCGCCGATCTTGTCAATG |
| HOXA5-Bisulf 1F | GTTAATTTTTTTTTGTTGTTGATGTGG |
| HOXA5-Bisulf 1R | CACGAATTTACCTCTAAAAATCATCAAAC |
| HOXA5-Bisulf 3F | GGTTTTTTGTAGTTATAGAGGTTGTTTGG |
| HOXA5-Bisulf 3R | AAACCCCAACAAAACCCAATCTC |
| HOXA4-Bisulf F | TATGGATTTTTTTTATTTAGGGG |
| HOXA4-Bisulf R | CCCAAATTCCCTCCCTTC |
| EYA4-Bisulf F | GTATTTCGTTTTTATTTGGTAGAGG |
| EYA4-Bisulf R | AACTACAAAAAACATTCAATCCAC |
| GATA2-Bisulf F | GGTAGAAATTTTGTGGGTTTTAGATT |
| GATA2-Bisulf R | TCTACACCCAAACCCTAAACC |
| HOXA11-Bisulf F | AGGTTAAGAATTATGTTGGGGTTTT |
| HOXA11-Bisulf R | AACTAAACACATCCTCTCCTCTCTC |
| HOXA13-Bisulf F | AAGAGTTTTTGATTTTTTTGGTTGA |
| HOXA13-Bisulf R | AAAAACCTTAAACCTTAACCTTAAC |
| RUNX1-Bisulf F | TAGGGTTAGGGTGTTGTGGT |
| RUNX1-Bisulf R | AAACAACTTCCTATCTTTTATCCTC |
| CDKN1-Bisulf F | AGGAGGGAAGTGTTTTTTTGTAGTA |
| CDKN1-Bisulf R | ACAACTACTCACACCTCAACTAAC |
| mH19-12- Bisulf F | GTAAGGAGATTATGTTTTATTTTTGG |
| mH19-12- Bisulf R | AAAAAAACTCAATCAATTACAATCC |
| mH19-34- Bisulf F | GGTATAGAAGTTGTTATGTGTAATAAGGG |
| mH19-34- Bisulf R | CCTAAAATACTCAAAACTTTATCACAAC |
| RT HOXA5 F | GCAAGCTGCACATAAGTCATG |
| RT HOXA5 R | AGGTAACGGTTGAAGTGGAAC |
| RT RUNX1_short F | TGTTTGCAGGGTCCTAACTC |
| RT RUNX1_short R | AACGCCTCGCTCATCTTG |
| RT RUNX1_long F | ACTTGGAATGAATCCTTCTAGAGAC |
| RT RUNX1_long R | CAACGCCTCGCTCATCTT |
| RT ActB F | TGTATGAAGGCTTTTGGTCTCC |
| RT ActB R | GTCTCAAGTCAGTGTACAGGT |
| ChIP-HOXA11 F | CAAAGGCCAAGAATCATGCTG |
| ChIP-HOXA11 R | GGGTTTTGCAGCGATTTCTTAG |
| ChIP-HOXA13 F | AGCTCTTGATTCCCTTGGTTG |
| ChIP-HOXA13 R | GTGACCTTGACTTTTGACAGC |

| | |
|---|---|
| ChIP-RUNX1 F | ACTGGCGTGGAGGCCTTTC |
| ChIP-RUNX1 R | TATCCTCGAACGCTAGTTCTTCC |
| ChIP-CDKN1 F | CTGCAGCACGCGAGGTT |
| ChIP-CDKN1 R | ACAAGGAACTGACTTCGGCAG |
| ChIP-HBB F | ATAAGATGGCAGGAGAGGTTTG |
| ChIP-HBB R | TTACTCAAGGAAAGGCAGTGG |
| Mut-C141S F | CTACAGCTTCCCTTCCCAGGACCTGAGCCAG |
| Mut-C141S R | CTGGCTCAGGTCCTGGGAAGGGAAGCTGTAG |
| Mut-Q147L F | GGACCTGAGCCAGCTAGGCATCCAGAAGGG |
| Mut-Q147L R | CCCTTCTGGATGCCTAGCTGGCTCAGGTCC |
| Mut-S317A F | ACACTGACAGCTGCCGGCGCCAACTCC |
| Mut-S317A R | GGAGTTGGCGCCGGCAGCTGTCAGTGT |
| HOXA5_off_#1 F | GTTTATTTGGTAAAGGATTGAGTAG |
| HOXA5_off_#1 R | AACTCTCAAACCCCTTACTTCTC |
| HOXA5_off_#2 F | GTTTGTGGGTTATTTGAATTGTTTT |
| HOXA5_off_#2 R | ATCAAACATTACAACCCTATCTTCCT |
| HOXA5_off_#4 F | ATAAGATTAGAGGTTGGGGTAAGAAG |
| HOXA5_off_#4 R | AAAATCCAACCTATACCAAAC |
| HOXA5_off_#5 F | TTGGATTTGTGATTGTAGTTGG |
| HOXA5_off_#5 R | CCCCACCTTCCACCC |
| HOXA5_off_#6 F | TAGGTTGTGGTATTGGTGGTATTG |
| HOXA5_off_#6 R | TAACATAAAAACCCCATTACAATTC |
| HOXA5_off_#8 F | GGTTTGGAGATATTTGATAGGTTTG |
| HOXA5_off_#8 R | AAAATAAAAAAACAAAAACAACTTCC |

**Supplementary Table 3. Statistics on the reduced representation bisulfite sequencing data.**

| Samples | Sequencing Reads | Mapped read percent | BS Conversion | Coverage CG Percent | Sequencing Depth | Methylation level |
|---|---|---|---|---|---|---|
| dCas9-MQ1$^{C141s}$ D1 | 9846543 | 72.50% | 99.30% | 10.30% | 18.1 | 0.51 |
| dCas9-MQ1$^{C141s}$ D2 | 8878012 | 80.30% | 99.30% | 11.00% | 21.1 | 0.51 |
| dCas9-MQ1$^{C141s}$ D4 | 13074060 | 82.60% | 99.30% | 11.10% | 30.9 | 0.52 |
| dCas9-MQ1$^{Q147L}$ D1 | 7476567 | 77.10% | 99.30% | 10.40% | 19.0 | 0.51 |
| dCas9-MQ1$^{Q147L}$ D2 | 12203837 | 81.30% | 99.30% | 11.10% | 29.2 | 0.51 |
| dCas9-MQ1$^{Q147L}$ D4 | 6026290 | 81.20% | 99.30% | 10.20% | 15.7 | 0.51 |
| dCas9-MQ1 D1 | 5053176 | 74.50% | 99.30% | 10.00% | 13.4 | 0.56 |
| U.C. D1 | 9912281 | 83.50% | 99.30% | 11.10% | 23.6 | 0.51 |
| U.C. D4 | 11239519 | 82.00% | 99.30% | 10.60% | 22.9 | 0.51 |
